# Prior knowledge about events depicted in scenes decreases oculomotor exploration

**DOI:** 10.1016/j.cognition.2023.105544

**Published:** 2023-07-05

**Authors:** Marek A. Pedziwiatr, Sophie Heer, Antoine Coutrot, Peter Bex, Isabelle Mareschal

**Affiliations:** a School of Biological and Behavioural Sciences, Queen Mary University of London, Mile End Road, London E1 4NS, United Kingdom; b Univ Lyon, CNRS, INSA Lyon, UCBL, LIRIS, UMR5205, F-69621 Lyon, France; c Department of Psychology, Northeastern University, 107 Forsyth Street, Boston, MA 02115, United States of America

**Keywords:** Eye movements, Visual perception, Prior knowledge, Scene viewing

## Abstract

The visual input that the eyes receive usually contains temporally continuous information about unfolding events. Therefore, humans can accumulate knowledge about their current environment. Typical studies on scene perception, however, involve presenting multiple unrelated images and thereby render this accumulation unnecessary. Our study, instead, facilitated it and explored its effects. Specifically, we investigated how recently-accumulated prior knowledge affects gaze behavior. Participants viewed sequences of static film frames that contained several ‘context frames’ followed by a ‘critical frame’. The context frames showed either events from which the situation depicted in the critical frame naturally followed, or events unrelated to this situation. Therefore, participants viewed identical critical frames while possessing prior knowledge that was either relevant or irrelevant to the frames’ content. In the former case, participants’ gaze behavior was slightly more exploratory, as revealed by seven gaze characteristics we analyzed. This result demonstrates that recently-gained prior knowledge reduces exploratory eye movements.

## 1. Introduction

Human experience of visual-information intake is continuous in time (Wurtz, Joiner, & Berman, 2011) and encompasses the ongoing transition from the past to the present (Hogendoorn, 2022). At any given moment, information about recent events is necessary for individuals to control their behavior (Tatler & Land, 2011). While some of that information might remain available in in the environment (for example, in the form of results of completed actions), much of it must be remembered. Here, we recorded people’s eye movements to investigate how the memory of the recent-past, or – in other words – recently-gained prior knowledge, influences humans’ ongoing exploration of their surroundings.

While it is known that recent experiences influence the processing of previously unviewed static stimuli across a range of tasks (Awh, Belopolsky, & Theeuwes, 2012; Fischer & Whitney, 2014; Horstmann, 2015; Loschky, Larson, Smith, & Magliano, 2020), their effects within the context of the perception of novel, complex naturalistic scenes remain underexplored. This is because in the vast majority of studies that involve viewing scenes and/or performing tasks related to them, each scene constitutes a separate, self-contained trial, independent of the other trials (see Tatler, Hayhoe, Land, & Ballard, 2011 and Dorr, Martinetz, Gegenfurtner, & Barth, 2010 for further critique of such studies). Consequently, such experimental settings inevitably render the accumulation of information across different, consecutively viewed scenes unnecessary. Yet, in the real world, where the visual input reaching human eyes usually has a continuous temporal structure and carries information about events that unfold over time, the accumulation of information is vital (Fairhall, Albi, & Melcher, 2014; Harrison, Bestmann, Rosa, Penny, & Green, 2011). Therefore, most laboratory-based studies involving image viewing do not tap into this process despite its importance and ubiquity in everyday life (with Hutson, Chandran, Magliano, Smith, & Loschky, 2022 being a notable exception). The goal of the present study was to address this gap and investigate how the information about recent past events, accumulated while observing their temporal development, influences human eye-movements.

In this preregistered study, we investigated whether identical images of scenes, presented under identical task conditions, can elicit different viewing behaviors depending on the content of the images immediately preceding them. All our experiments followed a similar design. In each trial, participants viewed a sequence of film frames extracted from films directed by Alfred Hitchcock. We created two experimental conditions. In both conditions, the final frames in the sequences, called the ‘critical frames’, were the same. The conditions differed only with respect to the frames presented prior to them, called the ‘context frames’. In one condition, the context frames were frames preceding the critical frame in the film from which it was extracted. In the other condition, the context frames came from a different film than the critical frame. Therefore, depending on the condition, the same critical frame depicted events that were either a natural continuation of the events from the previous frames or were unrelated to them. Consequently, in the latter condition, the prior knowledge about the recent past was useless for identifying image regions that do not need to be attended because they contain information that has already been acquired. We hypothesized that this would lead to more exploratory viewing behavior than in the former condition, where prior knowledge could guide the gaze. To preempt our findings, our hypothesis was confirmed. We found that identical stimuli (i.e. critical frames) were sampled differently, depending on the information participants acquired before viewing them. Specifically, we analyzed seven metrics characterizing oculomotor behavior and found differences related to the presence of relevant prior knowledge in six of them. This result demonstrates that prior knowledge about the recent past contributes to the control of ongoing oculomotor behavior.

### Open practices statement.

This study was preregistered. The preregistration protocol is available from the Open Science Framework repository (https://osf.io/et7mr/?view_only=6f86dc8211d845c7b2c09ef6f45baf64), together with the data from all experiments and a script for downloading our stimuli.

## 2. Experiment 1

**Fig. 1.**
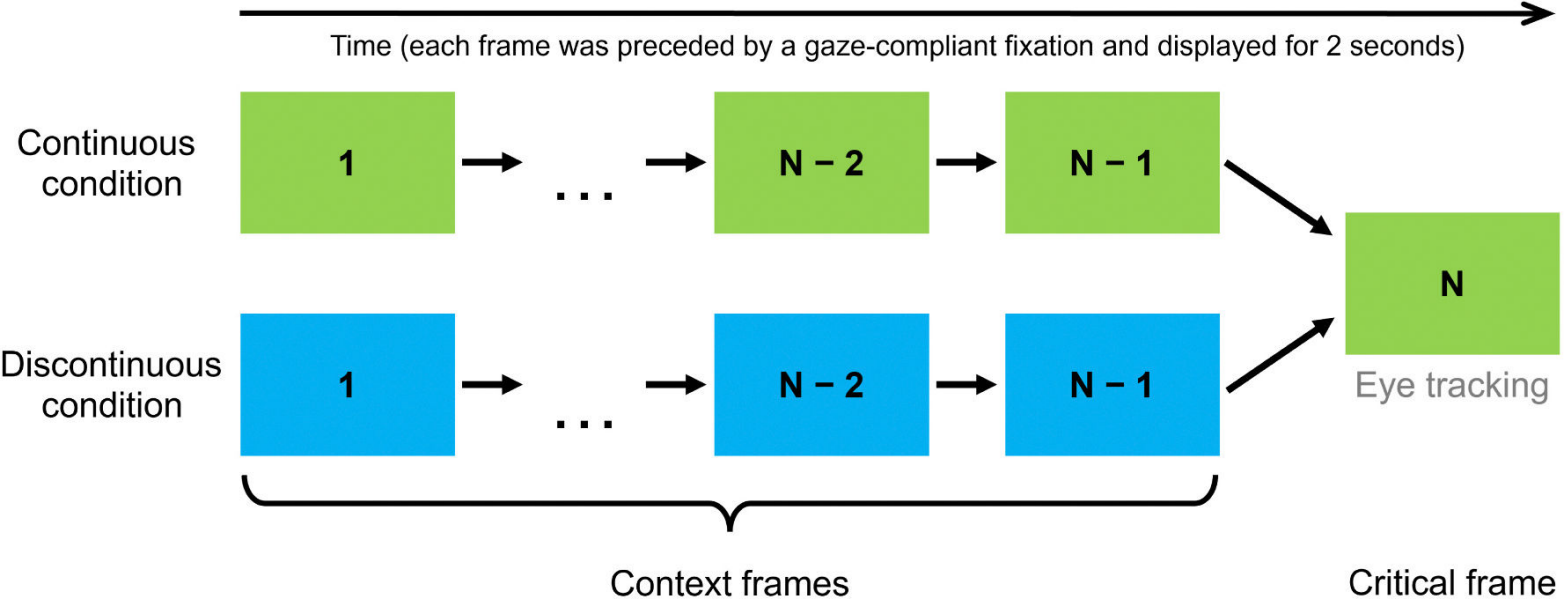
Experimental design. *Note.* Different colors indicate different films. The dots indicate that the number of context frames could differ between sequences (but for each different critical frame the number of context frames preceding it was identical in both conditions).

Forty-eight participants viewed sequences of film frames (static images taken from films). In each N-frame sequence, frames from N to N-1 served as a ‘context’ for the last frame, called a critical frame (see Fig. 1). In one condition, the context frames came from the same film as the critical frame and depicted a continuous (coherent and uninterrupted) course of events that naturally preceded events from the critical frame (Continuous condition). In the other condition, the context frames also depicted a continuous course of events but, crucially, came from a different film and therefore were unrelated to the critical frame (Discontinuous condition). Each participant viewed all critical frames but – given that the conditions were counterbalanced between two groups of participants – half of the frames were presented to them in the Continuous condition and the other half in the Discontinuous condition. None of the critical frames were ever used as context frames and vice versa. Consequently, none of the frames were presented more than once to the same participant. To ensure that participants remained attentive throughout the experiment, we presented an attention-check question after each critical frame (participants were asked: ‘*Which description best matches the last image?*’) and they had to select the correct description of the image they had just seen out of four descriptions presented, for example a) ‘*men in a room with a woman holding a gun*’, b) ‘*a group of people in an elevator*’, c) ‘*people walking down a hallway*’, d) ‘*a group of men looking through a stack of documents*’. Please refer to our Supplemental Materials for all questions used in our study. We hypothesized that in the Discontinuous condition, where prior knowledge about events from context frames was irrelevant to the content of the critical frame, participants would exhibit a more exploratory viewing behavior.

### 2.1. Method

#### Participants and sample size.

Forty-eight participants (40 women, mean age: 22 years) took part in the experiment, which is approximately twice the number of participants used in three similar studies (Coco, Nuthmann, & Dimigen, 2020; End & Gamer, 2019; Walter & Bex, 2021). Participants were all over the age of 18 and were predominantly staff and students of Queen Mary University of London. They all reported having normal or corrected to normal vision, provided written informed consent prior to their participation, and received remuneration (course credits or cash) afterwards. If a participant responded correctly to fewer than 80% of the attention-check questions or if a large amount of their gaze data was missing, we excluded them and tested another person instead (there were 5 such cases, see Excluding participants section in Supplemental Materials for details). All our experimental procedures were approved by the Queen Mary University of London research ethics board (QMERC20.307) and the Institutional Review Board of Northeastern University (#14–09-16).

#### Stimuli.

We used film frames from the “1000 frames of Hitchcock” website (https://the.hitchcock.zone/wiki/1000_Frames_of_Hitchcock), which stores frames from all films directed by Alfred Hitchcock (1000 per film). The frames are spaced several seconds apart and were extracted using a semi-automatic procedure involving human inspection to ensure that they were not – according to the creator of the website – ‘*blurred or looking odd’*.

We manually selected 936 frames that constituted our stimuli set. 80 of them were the ‘critical frames’. Each was paired with two sequences of ‘context frames’ that presented different, but always consistent and logical, courses of events: Continuous and Discontinuous. Continuous context-frames were frames preceding the critical frame in a film from which it originated. Therefore, each critical frame was a natural continuation of the context frames. Discontinuous context-frames came from a different film and were unrelated to the critical frame. Camera position and focus could change from frame to frame within a sequence. Context lengths varied across critical frames (M = 5.35 frames, SD = 1.16) but not within them: contexts paired with a given critical frame always had equal lengths in the two conditions. All frames were in color and had 16:9 aspect ratio. None of them contained violent or sexual content and none of the critical frames depicted a single person. We estimated that in the films from which the frames originated, the average time between two consecutive extracted frames amounted to 7.29 s (SD = 0.72). The number of critical frames was constrained by the amount of available stimulus material. For each critical frame, we created four descriptions of its content, of which only one was correct. These served as response options in the attention-check question presented after each critical frame.

##### Stimuli set validation.

We validated our stimuli set in two online experiments (see Supplemental Materials for details). Validation Experiment V1.1 confirmed that naïve participants could tell whether critical frames depicted a natural occurrence of events following from the context frames (for the Continuous contexts) or did not (for the Discontinuous contexts). Validation Experiment V1.2 confirmed that the attention-check questions were suitable (that is, sufficiently easy to answer). We iteratively modified our stimuli set (selected frames and question response options) and re-ran both experiments until, for each critical frame in both experiments, at least 8 out of 10 participants responded correctly.

#### Procedure and design.

The experiment lasted approximately 45 minutes and consisted of four blocks of 20 trials, interleaved with breaks. In each trial, participants freely viewed a sequence of frames (context frames followed by a critical frame) and then responded to the attention-check question. Half of the sequences viewed by each participant belonged to the Continuous condition, half to the Discontinuous (counterbalanced between groups to which the participants were assigned using an ABAB schema). The assignment of sequences to blocks was randomized for each participant.

Before starting the experiment, participants received verbal and onscreen instructions that stated (a direct quote): ‘*You will be presented with a sequence of images. Please look at each of them carefully.*’ and were informed about the presence of the attention-check questions. Then, they completed a sample trial. Before each block, the eye tracker was calibrated. All frames were presented for two seconds and participants’ eye-movements were recorded during that time. Prior to each frame, a gaze-compliant fixation dot appeared centrally on the screen. Participants had to fixate within a 1.5 degrees’ radius from it for 750 ms for a frame to be displayed. All critical frames were followed by the attention-check question ‘*Which description best matches the last image?*’ and four possible answers, out of which one had to be selected by pressing a corresponding button (‘d’, ‘f’, ‘g’, or ‘h’, assigned ‘a’, ‘b’, ‘c’ or ‘d’ with a sticker corresponding to the response choice) on a keyboard.

#### Apparatus.

The experiment took place in a dimly lit room. The frames were presented on a computer monitor against a grey background and had a height of 16 degrees of visual angle and a width of 28.4, which corresponded to 620 and 1102 pixels, respectively. Participants sat 55 cm from the monitor (iiyama Vision Master Pro 510; type: CRT; resolution: 1600 × 1200; refresh rate: 60 Hz; diagonal: 19 in.; brightness and contrast were adjusted to ensure comfortable viewing for prolonged times), with their heads stabilized in a chin rest. Their gaze was recorded using an infrared, video-based eye tracker Tobii 4C (90 Hz sampling rate). The procedure was programmed in MATLAB, using Psychophysics Toolbox Version 3 (Kleiner et al., 2007) and Tobii Pro Software Development Kit for Matlab version 1.7.1.4.

#### Oculomotor metrics.

We examined the following seven metrics of oculomotor behavior: the number of fixations, average fixation duration (measured in milliseconds), average inter-fixation distance (Euclidean distance between subsequent fixation points measured in degrees of visual angle, akin to saccade amplitude), inter-observer consistency (the similarity between the heatmap created from fixations of a given participant and the heatmap created from fixations of all remaining participants, determined by using a correlation between these heatmaps; Lyu et al., 2020), the probability of blinking (in our data, zero indicated no blinks, one – at least one blink), first-saccade latency (measured in milliseconds), and heatmap entropy (a measure of dispersion of all fixations registered on an image with values ranging from zero to one; Gameiro, Kaspar, König, Nordholt, & König, 2017). Apart from the entropy, that requires aggregating data from all participants viewing the same frame to calculate a single value, all metrics had unique values per participant per frame. The details of data pre-processing (including fixation-extraction procedure) and metrics calculation are provided in Supplemental Materials.

#### Data analysis.

We used functions from the R (R Core Team, 2020) package lme4 (Bates, Mächler, Bolker, & Walker, 2015) to analyze how our metrics differed between the Continuous and Discontinuous conditions. Specifically, we fitted (generalized) linear mixed-effects models (GLMMs and LMMs) to the data. All our models included an intercept and a fixed effect of experimental condition. We used LMMs (fitted using function *lmer*) for all metrics but the probability of blinking, for which we used a GLMM (logistic regression model fitted using function *glmer*). Models’ random effects structures included random intercepts both per frame (for all metrics) and per participant (for all metrics but the entropy, for which the data were aggregated over participants).

To establish which metrics differed between conditions in a statistically significant fashion, we followed guidelines provided by Brown (2021). Specifically, we tested for which metrics our models provided better fits to the data than models that did not include the fixed effects of the experimental condition but were otherwise identical. To this end, we used likelihood-ratio tests implemented in R function *anova*. When reporting the results, we provide beta coefficients from our models and their standard errors, as well as the values of a test statistic χ^2^ (always for one degree of freedom) and *p*-values from the likelihood-ratio tests. In our data, the Continuous condition was always coded as 0 and Discontinuous as 1. Therefore, in each LMM, beta coefficient for the intercept is an estimate of metrics value in the Continuous condition, while beta coefficient for the condition provides the estimate of difference between the two conditions.

### 2.2. Results

**Fig. 2.**
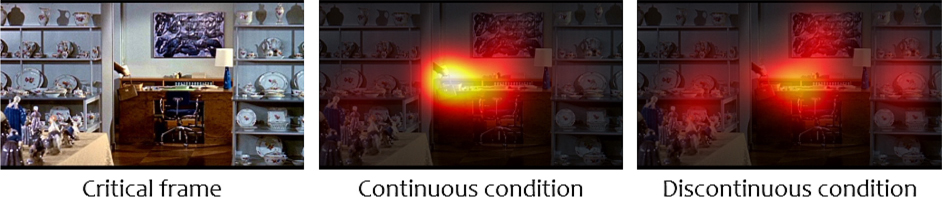
Sample critical frame and the heatmaps of fixations registered on it in different conditions in Experiment 1. *Note.* Each heatmap illustrates fixations of 24 unique participants overlaid on the critical frame and smoothed using a Gaussian kernel – see details in Supplemental Material. Pixel values in both heatmaps were jointly normalized, so their color values are directly comparable. The frame was taken from the film Topaz (Hitchcock, 1969).

We found statistically significant effects of condition for five out of our seven oculomotor metrics (see Table 1 for full results, Fig. 2 for sample heatmaps created from fixations recorded in both conditions, and Fig. S1 in Supplemental Materials for plots). Specifically, we found that in the Discontinuous condition, the number of fixations increased, average fixation duration decreased, average inter-fixation distance increased, inter-observer consistency increased, and the probability of blinking decreased. The two metrics for which the differences between conditions failed to reach the threshold of statistical significance were first-saccade latency and heatmap entropy.

### 2.3. Discussion

In Experiment 1 we tested the hypothesis that eye movements performed while viewing a scene are influenced by knowledge acquired earlier (prior knowledge). To this end, we analyzed gaze patterns of participants viewing identical images (the critical frames) in two conditions: Continuous, in which events depicted in the critical frames naturally followed from the events shown in the ‘context’ frames presented before, and Discontinuous, in which there was no relationship between the critical frames and the context frames. We found that the conditions differed for five out of seven oculomotor characteristics that we analyzed. The directions of these differences suggested that, in the Continuous condition, participants explored the critical frames less extensively than in the Discontinuous condition.

However, a potential concern regarding Experiment 1 is that each critical frame was followed by the attention-check question. Specifically, while the occurrence of the question could be anticipated in the Discontinuous condition – because it always appeared after a frame belonging to a different film than the previous frames in a sequence – it could not be anticipated in the Continuous condition, since in this condition all frames in a sequence belonged to the same film. Therefore, participants viewing critical frames in the Discontinuous condition could be changing their viewing strategy solely because they anticipated a question about the scene content afterwards. To exclude the possibility that this anticipation of the question fully explains the change in visual exploration, we conducted two more experiments. One is reported below as Experiment 2 (note that it was preregistered as Experiment 3), while the other experiment (preregistered as Experiment 2) is reported in Supplemental Materials as Experiment S1.

## 3. Experiment 2

Experiment 2 used the same the design as Experiment 1 except that the attention-check questions could appear after *any* frame, including the context frames. This ensured that the critical frames in the Discontinuous condition were no longer predictive of the occurrence of questions. This new design necessitated using different questions than in Experiment 1 (see below). The difference, if it had any noticeable effect at all, affected both conditions equally and therefore was unlikely to affect the differences between them (which were our primary output of interest).

### 3.1. Method

#### Participants and sample size.

We collected data from 50 participants (two more than planned; 29 women, mean age: 20.22 years). 44 were undergraduate students of Northeastern University. The remaining 6 belonged to the participant pool from which the participants for Experiment 1 were recruited. All participants declared having normal or corrected to normal vision. For the participants recruited at Northeastern University, this was confirmed using standard tests of visual acuity (ETDRS, Ferris & Bailey, 1996), color vision (H-R-R, Hardy, Rand, & Rittler, 1954), and stereo acuity (Titmus, Ohlsson et al., 2001). All excluded participants (4 cases) were replaced; see Excluding participants section in Supplemental Materials for details.

#### Stimuli.

We used the same 80 frame-sequences as in Experiment 1 and a novel set of attention-check questions. Specifically, we created 16 ‘generic’ multiple choice attention-check questions that could be asked after any frame and still have one unambiguously correct answer (for example: ‘*Is this scene likely to be in a city?*’; ‘*yes*’/’*no*’). Their full list is available in the Supplemental Materials.

Next, we paired the questions with our sequences (by a ‘sequence’ we mean a critical frame and two equinumerous sets of context frames, used in our Continuous and Discontinuous conditions). First, of the 60 sequences not used in Experiment S1, we randomly selected 48 sequences and paired each of them with one question, that could randomly occur after any context frame. Therefore, each of the 16 unique questions was used with three different sequences (out of the 60). Note that although the position of that frame in the context was the same in both conditions (e.g. the question appeared after a third frame), the actual image (frame) differed between the Continuous and Discontinuous conditions. Second, we randomly selected 16 out of the remaining 20 sequences and paired each with a unique generic question. In these sequences, the question was always presented at the very end, after a critical frame. In total, we created 112 unique frame-question pairs (16 × 3 × 2 + 16) which were the same across all participants. We validated these pairs in Validation Experiment V2.1, described in Supplemental Materials.

#### Procedure and design.

Experiment 2 differed from Experiment 1 regarding the content of the questions and their placement within the procedure: here, they could also appear after context frames. Additionally, to make the procedure more engaging, we shortened the duration of the gaze-compliant fixation dot preceding all frames from 750 ms to 375 ms. Apart from these two changes, Experiment 2 was identical to Experiment 1.

#### Apparatus.

The data were collected in two laboratories: at Queen Mary University of London, where the data for Experiment 1 were collected, and at Northeastern University in Boston. The setups in both laboratories were equivalent and used the same eye tracker model.

### 3.2. Results

Data pre-processing and analysis are the same as in Experiment 1. The results of Experiment 2 (see Table 2 and Fig. S2 in in Supplemental Materials) mirror the results of Experiment 1 for all metrics apart from two: the probability of blinking and first-saccade latency. Specifically, we found statistically significant differences between conditions for the number of fixations, average fixation duration, average inter-fixation distance, inter-observer consistency and first-saccade latency but not for the probability of blinking and heatmap entropy. Importantly, except for the entropy, all the metrics showed the same directions of differences between our conditions in both Experiments, irrespective of the statistical significance of these differences.

We analyzed aggregated data from both Experiments to check if they indeed differed qualitatively regarding the probability of blinking and first-saccade latency. To this end, we extended the (G) LMMs used for these metrics by including a main effect of experiment number (1, coded as 0, vs. 2, coded as 1) and an interaction between the experiment number and the experimental condition. Next, we used likelihood-ratio tests to check if these models provided better fit to the data than models without each of these effects. Whenever such test indicated that the initial model is better, this meant that the removed effect contributed to the quality of fit – this is how we determined the statistical significance of each effect. For the probability of blinking, all fixed effects were statistically significant (intercept: beta = −1.06, SE = 0.19; condition: beta = −0.32; SE = 0.08, χ^2^ (2) = 15.78, *p* < 0.001; experiment: beta = 0.41, SE = 0.26, χ^2^(2) = 10.34, *p* = 0.006; interaction: beta = 0.27, SE = 0.11, χ^2^ (1) = 5.72, *p* = 0.017), indicating that the drop in the probability of blinking between the Continuous and Discontinuous conditions was larger in Experiment 1 than in Experiment 2. For the first-saccade latency, we did not find statistically significant effects other than a decrease in the Discontinuous condition (intercept: beta = 323.03, SE = 14.18; condition: beta = −8.47, SE = 5.21; χ^2^(2) = 7.17, *p* = 0.028; experiment: beta = −26.72, SE = 18.06, χ^2^(2) =2.64, *p* = 0.268; interaction: beta = −2.53, SE = 7.34, χ^2^(2) = 0.12, *p* = 0.730).

## 4. Discussion

Our study investigated how prior knowledge about recent events affects gaze control. Participants viewed the same film frames, called the critical frames, while possessing prior knowledge either about events leading to situations depicted in these frames (in the Continuous condition), or about unrelated events (in the Discontinuous condition). We analyzed seven characteristics of eye movements registered on these frames and found differences between conditions for six of them (the number of fixations, average fixation duration, average inter-fixation distance, inter-observer consistency, the probability of blinking, and first-saccade latency). These differences, although small, indicated that in the Continuous condition, visual processing was more focused on key elements, while in the Discontinuous, it was more exploratory. Our results therefore demonstrate the role of prior knowledge about the recent past in oculomotor control. Importantly, given that this knowledge is rapidly acquired (as our results show) and usually, albeit not always, short lived (Hannula, 2010; Hollingworth & Henderson, 2002; Irwin & Zelinsky, 2002), it is distinct from a general knowledge about the world which, for example, allows for identifying inconsistent objects within scenes, such as an octopus in a farmyard (Loftus & Mackworth, 1978; see also Smith, Loschky, & Bailey, 2021).

When comparing participants’ gaze behavior between conditions, in the Discontinuous condition we observed a slight increase in the number of fixations and in the inter-fixation distance, as well as a small decrease in fixation duration. These changes in gaze behavior – apart from indicating that observers explored the scenes somewhat more – might indicate a shift in the mode of visual processing from focal (focused on examining restricted image regions in detail) to ambient (focused on grasping the overall layout of a scene. The distinction between these two processing modes (or at least viewing modes) has a history dating back to 1947 – see (Pannasch, Helmert, Roth, Herbold, & Walter, 2008) for a brief summary. Although the precise neural underpinnings of ambient and focal processing are still being debated (Marsman., 2013; Mills et al., 2017), the distinction itself has been demonstrated using tasks such as simulated driving (Velichkovsky, Rothert, Kopf, Dornhöfer, & Joos, 2002), solving Rubik’s cube (Guo, Helmert, Graupner, & Pannasch, 2022), film viewing (Eisenberg & Zacks, 2016), and viewing scenes of different kinds (Pannasch et al., 2008; Unema, Pannasch, Joos, & Velichkovsky, 2005), as well as with computational modelling (Follet, Fontaine, & Meur, 2011; Le Meur & Fons, 2020). One well established behavioral finding related to this distinction is that when individuals view static scenes, their gaze behavior changes from ambient to focal over time (note that this is in opposite direction to the change we observed between our Continuous and Discontinuous conditions). For example, Pannasch et al. (2008) found that the first two seconds of scene viewing are characterized by more ambient processing than the seconds from four to six. The effects we found, although smaller, were obtained for images viewed for two seconds. Therefore, they extend the findings of Pannasch and colleagues by suggesting that the initial balance between ambient and focal processing is influenced by prior knowledge. Specifically, the initial processing is more focal if the prior knowledge is relevant to the viewed situation (see also Eisenberg & Zacks, 2016). The decrease of the first-saccade latencies we also observed in the Discontinuous condition – amounting to shorter first fixations – indicates that the effect we investigated emerges soon after stimulus onset. This result dovetails with the observation that a process happening within a similar time window, namely, gist extraction, also can be affected by prior knowledge provided to participants in a similar fashion as in our experiment (McLean, Nuthmann, Renoult, & Malcolm, 2023; see also Smith & Loschky, 2019).

Interestingly, heatmap entropy – a metric previously used to quantify the tendency to explore an image (Gameiro et al., 2017; Shiferaw, Downey, & Crewther, 2019) – did not differ between our experimental conditions. Entropy value for a heatmap depends on its spread, which, in turn, depends on fixated image regions. The lack of changes in entropy suggests that in both conditions, largely the same regions of the critical frames were fixated. This was probably because our frames, including the critical ones, usually contained only several key regions (e.g., faces) that attracted the majority of fixations irrespective of the condition. The existence of such regions, together with the observed increase in the number of fixations, likely explains another effect we found in the Discontinuous condition: the slight increase in inter-observer consistency. It is likely that in this condition more participants fixated all the key image regions, thus decreasing the overall variability among fixation patterns. Both these results highlight caveats in using film frames as stimuli.

The last metric we analyzed was the probability of blinking. It decreased in the Discontinuous condition in both Experiments, suggesting heightened attention to the critical frames (Ranti, Jones, Klin, & Shultz, 2020; see also Nakano, Kato, Morito, Itoi, & Kitazawa, 2013). This decrease was larger in Experiment 1, where the critical frames in the Discontinuous condition were reliable predictors of the questions. The anticipation of questions likely strengthened the increase in attentiveness elicited by the critical frame alone, which was marked by the further decrease in the probability of blinking.

While our effects are interesting from a theoretical perspective, it is worth considering their practical significance too. Funder and Ozer (2019) proposed that when assessing effects in psychological experiments, there are at least two important factors to take into account. The first factor is the absolute size of the effect. The effects we found were small. There are, however, two caveats to consider here. First, they in part depended on the specifics of our stimuli, because at least some of the oculomotor characteristics we measured depend on stimuli content (Le Meur & Fons, 2020; Pannasch et al., 2008) and size (Gameiro et al., 2017; Marsman., 2013; Otero-Millan, Macknik, Langston, & Martinez-Conde, 2013). Second, establishing the relevance of effects found in laboratory-based eye tracking studies to real-world situations is challenging (Henderson, 2017; Tatler et al., 2011), and, therefore, it is hard to judge if the effects we observed would be correspondingly small in more ecologically valid settings. Nevertheless, even under the assumption that our effects are small, they could still have a practical significance because of the second factor proposed by Funder and Ozer (2019) – the frequency of the real-world situations in which a phenomenon of interest occurs. We investigated the influence of prior knowledge that is accumulated whenever humans view unfolding events on a behavior that is ubiquitous, namely, eye movements. Given how common these two phenomena are, even minor effects related to them are worthy of attention.

An intuitive explanation of our findings is that participants were surprised by the events shown in the critical frames only in the Discontinuous condition. This explanation has some merit, and we refer to the definitions of surprise to interpret our results. Although these definitions vary (Barto, Mirolli, & Baldassarre, 2013; Modirshanechi, Brea, & Gerstner, 2022; Reisenzein, Horstmann, & Schützwohl, 2019), many of them highlight two mutually non-exclusive cases when an event can be surprising. First, the event can be inconsistent with the individual’s current mental model of the world. In our study, the context frames in both conditions provided participants with knowledge enabling them to build such mental models (Loschky et al., 2020; see also Smith, 2012): situations presented in them were changing in a sensible fashion. For example, a man sitting in a car in one frame was shown next to the car in a next frame, which indicated that he had left the vehicle. In the Discontinuous condition, the events in the critical frames were breaking the logical continuity of what was happening in the context frames. Therefore, these events were discrepant with the mental models that were based on the knowledge accumulated while viewing the context frames and were therefore surprising in that sense.

The second case in which an event can be called surprising is when it disproves previously generated predictions. The mental models described above can likely be sources of predictions about future events. For illustration, consider that subsequent frames often depicted the same characters in the same settings, so it was possible to predict the reappearance of a character in subsequent frames. Moreover, the general familiarization with our stimuli could lead to expectations about what sort of events are likely to occur, and which are not. For example, the appearance of a man in a long, black coat was quite likely while the appearance of a giraffe was not. Therefore, each critical frame had the potential to either confirm predictions (to some acceptable degree) or disprove them, and whenever the latter was the case, the frame was surprising.

It is worth noting that the predictions generated in our procedure were likely less specific than those investigated in many previous studies focusing specifically on predictions (Ekman, Kusch, & de Lange, 2023; Kok, Failing, & de Lange, 2014; Kok, Jehee, & de Lange, 2012) and related phenomena, such as surprise (Horstmann, 2015) and expectations (Amit, Abeles, Carrasco, & Yuval-Greenberg, 2019). Typically, these studies use a limited number of stimuli, and each stimulus is often paired with a specific cue that precedes it. For example, a seminal study by Kok et al. (2012) used gratings that were tilted either to the right or to the left and the tilt directions were paired with different auditory cues preceding grating presentation, leading to specific predictions about the upcoming visual input. In contrast, in our study the breadth of possible visual inputs was much broader which likely resulted in less specific predictions. In fact, we speculate that these predictions were even less specific than the ones typically generated in everyday situations. This is because in everyday situations, many changes in an individual’s visual input result from self-motion (which is under the individual’s control, e. g. during walking) and in consequence can be predicted more precisely than changes resulting from the motion of a camera (like in our study). Therefore, our experimental approach is complementary to the one used by Kok and colleagues. This is because these two approaches focus on predictions that are, respectively, more and less specific than the ones formed in everyday situations.

Prior knowledge about the recent past, which underpins the mental models that facilitate prediction generation, must be stored in memory. The relationship between oculomotor control and different types of memory is intricate and while we demonstrate one way in which it manifests itself, there are many more (for reviews, see Hannula, 2010; Henderson, 2017; Van der Stigchel & Hollingworth, 2018; Wynn, Shen, & Ryan, 2019). For example, semantically inconsistent objects in scenes (like an octopus in a farmyard) are looked at differently than consistent objects that are matched for visual features and scene location (Coco et al., 2020; Öhlschläger & Võ, 2017). While this effect relies on a general knowledge about the world stored in the long term memory (for example, the knowledge that octopuses do not belong farmyards), we observed effects that are driven by knowledge that has been acquired only recently. While it is likely that both these forms of knowledge are commonly used in conjunction to guide human gaze, the exact nature of their interaction remains poorly understood.

Another interesting question related to the relationship between memory and eye movements stems from the fact that our results mirror the results from two recent studies investigating this relationship (Cronin, Peacock, & Henderson, 2020; Walter & Bex, 2021). These studies investigated how viewing scenes under high memory load differs from viewing scenes under low load and found differences in gaze behavior that echo the differences we observed between the Continuous and Discontinuous conditions. Given this similarity in results, it is tempting to consider if they can be driven by the same mechanism. Specifically, it is possible that the balance between the ambient and focal viewing modes is influenced by the current memory load – the higher the load, the less ambient the viewing (see Eisenberg & Zacks, 2016 for a similar suggestion). Although our study – unlike the two aforementioned ones – did not manipulate memory load directly, it still could be changing in it. This is because the critical frames in the Discontinuous condition could signal – already at the early stages of processing – that the currently upheld mental model is no longer relevant and can be removed from memory. This, in turn, might have decreased the memory load and lead to more exploratory behavior.

In our experiments, constructing mental models was one of the consequences of accumulating knowledge about the unfolding events. These events were conveyed by the semantic content of the frames. In typical scenes such as film frames, this content is inseparable from the visual features of stimuli (Pedziwiatr, von dem Hagen, & Teufel, 2023) – for example, human silhouettes indicate the locations of specific characters. Therefore, it is necessary to consider our findings in the context of an interplay between the frame-to-frame changes in visual features and prior-knowledge accumulation. In everyday situations, as well as in dynamic stimuli such as movie clips, visual features often change continuously because of the movement of scene elements such as objects or people. Motion strongly attracts eye movements (Itti, 2005; Mital, Smith, Hill, & Henderson, 2011; Roth, Rolfs, Hellwich, & Obermayer, 2023), and it has been suggested that in the presence of (object) motion, the knowledge accumulation is redundant for eye movements control (Wang, Freeman, Merriam, Hasson, & Heeger, 2012). Our stimuli were static and thereby rendered tracking any elements in a continuous fashion impossible (see a recent study by Hutson et al. (2022) for a similar approach). Nevertheless, our stimuli still did contain visual features that could be tracked and compared between the frames. Such tracking, however, is unlikely to fully explain our results. This is because several studies have demonstrated that even for dynamic stimuli in which continuous, motion-based tracking is possible, the narrative structure and semantic content of stimuli – which likely cannot be processed based on visual features alone (Pedziwiatr et al., 2023) – impact oculomotor control (Goettker, Pidaparthy, Braun, Elder, & Gegenfurtner, 2021; Kirkorian & Anderson, 2018; Loschky, Larson, Magliano, & Smith, 2015). Here, when such tracking was not possible, the reliance on the high-level factors must have been even stronger.

Taken together, we demonstrated that prior knowledge about recent past-events modulates the degree of ongoing oculomotor exploration during static-image viewing. Given that this type of knowledge is accumulated whenever individuals observe unfolding events, we contend that it is necessary to include it among factors routinely guiding gaze.

## Supplementary Material

Supplement

## Figures and Tables

**Table 1:** Results of Experiment 1.

Metric	Effect direction	Beta and SE for intercept	Beta and SE for condition	χ^2^(1)	p-value
Number of fixations	↗	5.25 (0.15)	0.41 (0.04)	86.07	<0.001 ***
Fixation duration (in milliseconds)	↘	308.41 (11.4)	−20.86 (3.8)	29.95	<0.001 ***
Inter-fixation distance (in degrees of visual angle)	↗	4.35(0.18)	0.32 (0.05)	45.35	<0.001 ***
Inter-observer consistency	↗	0.79 (0.01)	0.02 (<0.01)	22.81	<0.001 ***
Probability of blinking	↘	−1.05 (0.2)	−0.32 (0.08)	15.31	<0.001 ***
First-saccade latency (in milliseconds)	–	323.23 (16.45)	−8.78 (5.05)	3.02	0.082 n. s.
Heatmap entropy	–	0.54 (0.01)	0.01 (<0.01)	2.36	0.124 n. s.

*Note.* Asterisks in the last column indicate statistical significance and follow a standard convention. This convention is used in all tables in this article.

**Table 2:** Results of Experiment 2.

Metric	Effect direction	Beta and SE for intercept	Beta and SE for condition	χ^2^(1)	p-value
Number of fixations	↗	5.19 (0.13)	0.22 (0.05)	22.70	<0.001 ***
Fixation duration (in milliseconds)	↘	298.18 (9.67)	−12.47 (3.88)	10.31	0.001 **
Inter-fixation distance (in degrees of visual angle)	↗	4.41 (0.18)	0.2 (0.05)	14.62	<0.001 ***
Inter-observer consistency	↗	0.74 (0.02)	0.03 (<0.01)	34.32	<0.001 ***
Probability of blinking	–	−0.64 (0.18)	−0.06 (0.07)	0.59	0.442 n. s.
First-saccade latency (in milliseconds)	↘	296.51 (11.75)	−11.54 (5.28)	4.77	0.029 *
Heatmap entropy	–	0.56 (0.01)	−0.01 (<0.01)	1.79	0.181 n. s.

## Data Availability

Pease see Open practices statement section.

## Appendix A. Supplementary data

Supplementary data to this article can be found online at https://doi.org/10.1016/j.cognition.2023.105544.
