# Supplementary material for: Prior knowledge about events depicted in scenes decreases oculomotor exploration
Source: Cognition. Author manuscript; Available in PMC 2026 Mar 30. (PMC13035412; doi:10.1016/j.cognition.2023.105544)
Supplement: Supplement [file NIHMS2036176-supplement-Supplement.docx]

**Prior knowledge about events depicted in scenes decreases oculomotor exploration**

*Supplemental Materials*

Marek A. Pedziwiatr, Sophie Heer, Antoine Coutrot, Peter Bex, Isabelle Mareschal

**Validation Experiments V1 and V2**

The aim of these experiments was to ensure the suitability of the frame sequences we selected and our attention-check questions. All three experiments were implemented as Qualtrics (Qualtrics, Provo, UT) surveys administered via Prolific ([https://prolific.co](about:blank)). Participants were compensated at the rate of £7.50 an hour. They were adults who were naïve to the aims of these experiments and had Prolific approval rates of above 80%. Participants recruited for the Validation Experiments V1.1, V1.2, and V2.1 resided respectively, exclusively in the UK, in the UK or in the US, or exclusively in the US. We matched their countries of residence to the countries where we planned to collect the eye-tracking data. In all experiments, the order of presentation of items was randomized for each participant.

In the Validation Experiment V1.1 we tested whether naïve individuals could correctly identify whether the critical frame naturally followed from the sequence of context frames or not, that is, whether they could detect the Discontinuous sequences. Participants viewed the same sequence of frames as in the main experiment and were asked whether the last frame (the critical frame) ‘*appeared to be a natural continuation of the frames leading up to it*’ or not. Prior to starting, they were shown sample sequences from both the Continuous and the Discontinuous conditions.

We conducted the Validation Experiment V1.2 to ensure that the attention-check questions used in Experiment 1 were suitable. On each trial, participants viewed a critical frame for 2 seconds and then had to indicate ‘which description best matches the previous image’ by selecting one of four descriptions shown.

The aim of Validation Experiment V.2.1 was to test frames paired with the ‘generic’ attention-check question in Experiment 2 (see the next section). We used the same survey as for Validation Experiment V1.2. It was completed by 20 participants. Each participant responded to all our 112 frame-question pairs.

**Experiment 2 – frame-question pairs validation**

In order to set a minimum accuracy threshold for the ‘generic’ attention check questions that were paired with different frames in Experiment 2, we determined what accuracy we could reasonably expect. To this end, we conducted an online Validation Experiment V2.1 (see details above).

For each frame-question pair, we treated the most frequent response (the mode response) as correct. We confirmed this assumption by manually comparing responses to the frame content in several randomly-selected frame-questions pairs. Next, we calculated the percent of correct responses for each pair and discarded 33 frame-question pairs for which the accuracy was below 80%. We discarded these pairs because they were likely to be confusing, which is not surprising since the questions were generic and their pairing with frames was random.

Furthermore, we discarded 5 frame-question pairs that had more than 2 responses options and for which the average correct response rate was lower than a chance level multiplied by two. Additionally, we lowered the minimal acceptable percent of correct responses to the attention-check questions (that is, non-discarded frame-question pairs a given participant was presented with) to 70%. These decisions were made after we had looked at the rates of correct responses of several participants from Experiment 2 and realized that sometimes they were lower than expected based on Experiment 1. Presumably, this is because the validation experiment was much shorter (it did not include the contexts), so it was easier for participants to remain highly focused.

**Excluding participants**

In all three experiments (Experiments 1, 2 and S1), we excluded participants for whom data from excessively many critical frames had to be discarded (see next section). In Experiments 1 and S1, we also excluded participants who provided fewer correct answers to the attention-check questions than the thresholds we set. These thresholds were 80% in Experiment 1 and 70% in Experiment 2. The threshold was lower in Experiment 2 because the task was more difficult. The numbers of excluded participants amounted to 5 for Experiment 1, 4 for Experiment 2, and 0 for Experiment S1. We always recruited new participants to replace the excluded ones.

**Excluding data from a single participant for a single critical frame**

We discarded data from an individual participant viewing a single critical frame whenever at least one of the following four cases occurred. First, when the participant had difficulty fixating on the gaze-complaint fixation dot and the experimenter had to intervene for the experimental procedure to continue. Second, when the eye position was not recorded for more than 30% of image presentation time (for example due to an excessive amount of blinking). Third, when the eye position was not recorded for more than 30% of time when context frames for a given critical frame were being presented. Fourth, when only one fixation was recorded on a frame. Applying these criteria resulted in excluding 1.25%, 1.9%, and 2.71% of data from the Experiments 1, 2, and S1, respectively.

**Excluding first fixations**

When calculating heatmap entropy and inter-observer consistency, we discarded the first fixations. These metrics are particularly sensitive to the spatial distributions of fixations, so we wanted to avoid including fixations whose position could not be influenced by our experimental manipulation but was driven by the fixation dot that preceded the image. The first fixations were included in all the remaining analyses – which is a deviation from the preregistration – because we realized that excluding them is justified only in the two aforementioned cases only.

**Eye movements data: quality, pre-processing, heatmap creation, and metrics calculation**

All steps described in this section were conducted in MATLAB R2021a. In all analyses, we used data from the right eye only. The percentage of trials in which no data loss was observed (eye position has been recorded in all eye tracker samples) amounted to 68.98% in Experiment 1 (58.67% in Experiment 2). In the remaining trials, the average data loss was 12.46 samples (SD = 8.69) in Experiment 1 and 13.10 in Experiment 2 (SD = 8.56). Empirically determined sampling frequency (average number of samples registered per trial divided by the number of seconds in the trial) was 90.95 Hz (SD = 0.79) in Experiment 1 and 89.44 (SD = 0.75) in Experiment 2. In order to maximize (post hoc) the accuracy of gaze measurement, for data from each critical frame and each participant individually, we calculated a ‘drift-correction’ and, based on a visual data-inspection, decided to *not* apply it only in cases when it was unusually large, that is, exceeded 4 degrees. To calculate the correction, we assumed that the first 27 samples registered by the eye tracker on a frame (corresponding to the first 300 ms of viewing) were at the position of the fixation dot that preceded the image and we calculated a vector between the screen center and the average eye position during that period. Applying the correction involved shifting all data points in the trial by this vector. We used the same data to assess the accuracy of our eye trackers (that is, the difference between the actual gaze position and gaze position indicated by the eye tracker). We did it in two ways. First, we calculated the average magnitude of these drift correction vectors. In Experiment 1, it amounted to 0.71 degrees (SD = 0.41; median = 0.65; interquartile range = 0.45), while in Experiment 2 – to 0.82 degrees (SD = 0.53; median = 0.71; interquartile range = 0.69). Second, we calculated the average shift of the registered eye position away from the fixation along the horizontal and vertical dimensions. In Experiment 1, these values were -0.13 and -0.19 degrees, respectively. In Experiment 2, they amounted to -0.06 and -0.21 degrees. These measurements suggest that while our data was noisy, the systematic displacement between the measured eye position and the actual eye position was minimal. Fixation locations and durations were extracted from the eye-tracker data using the *fixation_detection* function from EyeMMV toolbox (Krassanakis et al., 2014). This function expects three parameters as arguments: one temporal (minimum duration threshold that we set to 50 milliseconds) and two spatial (t1, set to 20 pixels, and t2, automatically estimated by the function for each trial). Fixations that landed outside of the image boundaries were excluded from analyses. The inter-fixation distance was calculated as the Euclidean distance between sequential fixation points. Blinks were defined as periods of data loss lasting between 50 and 1000 milliseconds. First-saccade latency was defined as the interval between stimulus onset and a moment when eye velocity exceeded the threshold of 35 degrees per second. We did not analyze latencies shorter than 70 ms because such rapid saccades were most likely anticipatory.

In each condition, a single value of heatmap entropy was calculated per critical frame using a bootstrapping approach similar to the one used by Gameiro and colleagues (2017). We 1) randomly selected N_ent_ fixations from the available pool, 2) smoothed their discrete distribution using the MATLAB function *antonioGaussian* provided by Bylinskii (retrieved from https://github.com/cvzoya/saliency; its argument fc was set to 6), 3) calculated the entropy of the resulting heatmap using a MATLAB function *entropy*, and 4) divided the obtained value by the entropy of a uniform random distribution (for normalization). The final entropy value was obtained by averaging entropy values from 500 repetitions of steps 1) to 4). We tested different values of N_ent_ (seven, nine, and eleven) on the data from Experiment 1. They all yielded very similar vectors of entropy values (calculated for the whole dataset): all pairwise correlations between these vectors were higher than 0.98. In the manuscript, we always report results for N_ent_ = 9.

We calculated inter-observer consistency for each possible combination of participant, critical frame, and experimental condition using a standard leave-one-out procedure (Lyu et al., 2020). Specifically, for each participant, we quantified the similarity between the heatmap from their fixations and a heatmap from fixations of the remaining participants on each critical frame. The heatmaps were created using the same method as when calculating heatmap entropy. The similarity between them was calculated using a linear correlation coefficient.

For each participant in each trial, we checked if they blinked at least once and coded this information as one (blinks) or zero (no blinks). Because the information about blinks coded in this way was analyzed using a logistic regression model, we call this characteristic of gaze behavior the probability of blinking (see Nuthmann & Einhäuser, 2015 for a similar approach). We adopted this approach despite our preregistered plan to analyze the number of blinks because in Experiment 1 we found that in most trials no more than one blink occurred.

**Experiment S1**

**Experiment S1 – overview**

In Experiment 1, we found that the critical frames in the Discontinuous condition were looked at differently than in the Continuous condition. However, all critical frames were followed by attention-check questions but in the Discontinuous condition, it was possible to predict their occurrence (because they always appeared after a frame that originated from a different film than the frames presented before). Therefore, it is possible that participants changed their viewing behavior whenever they knew that the question would appear after the frame. To test if this process could explain the results of Experiment 1, we conducted Experiment S1 (preregistered as Experiment 2).

It was identical to Experiment 1 in all aspects but two. First, to eliminate the potential confound present in Experiment 1, Experiment S1 did not include any attention-check questions. Second, given that removing the questions removed the incentive for participants to remain engaged and therefore could have detrimental effects on their attentiveness, Experiment S1 contained only one block of 20 trials, instead of four blocks as Experiment 1. We assumed that with a shorter duration, the procedure would have been completed before participants lost their focus.

**Experiment S1 – stimuli**

Shortening the experiment fourfold required limiting the number of frame-sequences included in the procedure. To minimize the resulting decrease of statistical power, out of 80 critical frames (and their contexts) used in Experiment 1, we selected a subset of 20 for which – according to simple simulations described in the preregistration – the effects in Experiment 1 would be detectable with a much smaller sample. In our simulations, we used three out of seven characteristics of gaze behavior that we analyzed in Experiment 1: the number of fixations, average fixation duration, and average inter-fixation distance. Using only them was both logically justified and computationally feasible – see the preregistration for details.

**Experiment S1 – procedure and apparatus**

The procedure was the same as in Experiment 1, with three exceptions. First, it contained only one, uninterrupted block of 20 frame-sequences. Second, there were no attention-check questions after the sequences. Third, the duration of a fixation preceding the frames was set to 375 ms (as in Experiment 2). Data collection for this experiment took place in the same laboratory as for Experiment 1.

**Experiment S1 – participants and sample size**

We adopted a flexible approach to determining the sample size, that is, we decided to test up to 48 participants to match the sample size of Experiment 1 but analyzed data after testing 30 people and, given that we did not find statistically significant results, then tested a further 18 people. We would have stopped the data collection, had the results been different. We decided to continue data collection after analyzing the three metrics we used when selecting frame-sequences used in this experiment: the number of fixations, average fixation duration, and average inter-fixation distance.

Conducting such interim analysis increases the chance of false-positives when the alpha level (that is, a threshold of statistical significance) is not adjusted accordingly (Lakens, 2014). Here, we determined the alpha level using Pocock’s procedure (Pocock, 1977). It allows to determine how the alpha level should be adjusted when a researcher statistically analyses a part of data collected before reaching a planned number of data points and conditions further data collection on the result of this analysis. We relied on the implementation of this procedure provided in R package GroupSeq (the value of parameter ‘function’ set to ‘Exact Pocock Bounds’; Pahl, 2018) and obtained the following adjusted alpha levels: 0.031 for the interim analysis conducted on data from 30 participants and 0.019 for the final analyses reported here, in which we include data from 48 participants (39 women, mean age: 21.98). Please note that the purpose of these adjustments is to keep the effective alpha level for this experiment at 0.05.

**Experiment S1 – results**

We pre-processed and analyzed the data in the same way as for Experiment 1. The results are summarized in Table S1. The fixed effect of the experimental conditions was not statistically significant in any of the models fitted to the data. Note that here, the alpha level against which the reported p-values should be compared is 0.019 (see above).

**Table S1.** Results of Experiment S1.

| Metric | Beta and SE for intercept | Beta and SE for condition | χ2(1) | p-value |
| --- | --- | --- | --- | --- |
| Number of fixations | 5.04 (0.18) | 0.17 (0.1) | 3.11 | 0.078 n.s. |
| Fixation duration | 327.83 (15.48) | -15.16 (9.3) | 2.65 | 0.103 n.s. |
| Inter-fixation distance | 4.15 (0.22) | 0.24 (0.11) | 4.58 | 0.028 * |
| Inter-observer consistency | 0.7 (0.02) | 0.02 (0.01) | 2.52 | 0.112 n.s. |
| Probability of blinking | -0.82 (0.23) | -0.09 (0.16) | 0.29 | 0.591 n.s. |
| First-saccade latency | 355.19 (24.69) | -18.64 (13.24) | 1.98 | 0.159 n.s. |
| Heatmap entropy | 0.58 (0.01) | 0.002 (0.01) | 0.04 | 0.834 n.s. |

**Experiment S1 – discussion**

Experiment S1 – contrary to our expectations – yielded different pattern of results than Experiment 1. Specifically, we found statistically significant differences in gaze behavior between the experimental conditions only for the inter-fixation distance. This outcome could be interpreted in two ways: (1) that our effects of interest, at least for metrics other than the inter-fixation distance, are either non-existent or too small to be detected in our data and that the effects found in Experiment 1 are attributable solely to mechanisms triggered by the anticipation of question, or (2) that the effects of prior knowledge are the strongest when visual input that viewers receive remains highly relevant for them. This was the case in Experiment 2 (with the unpredictable questions) but not in Experiment S1, which did not offer any incentive for participants to remain attentive. The results of Experiment 2 support the second interpretation. Our effects are likely to emerge when participants are motivated to attend to the frames (and hence pay attention to the unfolding events). This, however, does not necessarily mean that they do not emerge when participants are not encouraged to pay attention. Indeed, it is possible that Experiment S1 had insufficient statistical power and we note that in this experiment we collected four times less data than in the other two experiments (since there were fewer sequences). Therefore, although it is noteworthy that the directions of effects in Experiment S1 are mostly the same as in the remaining experiments, the question whether the effects of prior knowledge are observable when participants are not strongly encouraged to pay attention remains open.

**Second analysis listed in the preregistration**

In our preregistration, we declared that we would analyze the data in two ways: using (generalized) linear mixed-effects models and using hidden Markov models. Here, we report the first analysis only. The second analysis will be reported in a separate article.

**Plots of results from Experiments 1 and 2**

Please note that the Y axes on different plots on Fig. S1 and Fig. S2 below start at different values. Asterisks indicating statistical significance are taken from Tables 1 and 2 in the main text. Also, note that we plot the number of blinks, not the probability of blinking we analyzed. ‘CIs’ in the legends of plots stand for confidence intervals.

**Figure S1.**

*Results of Experiment 1*


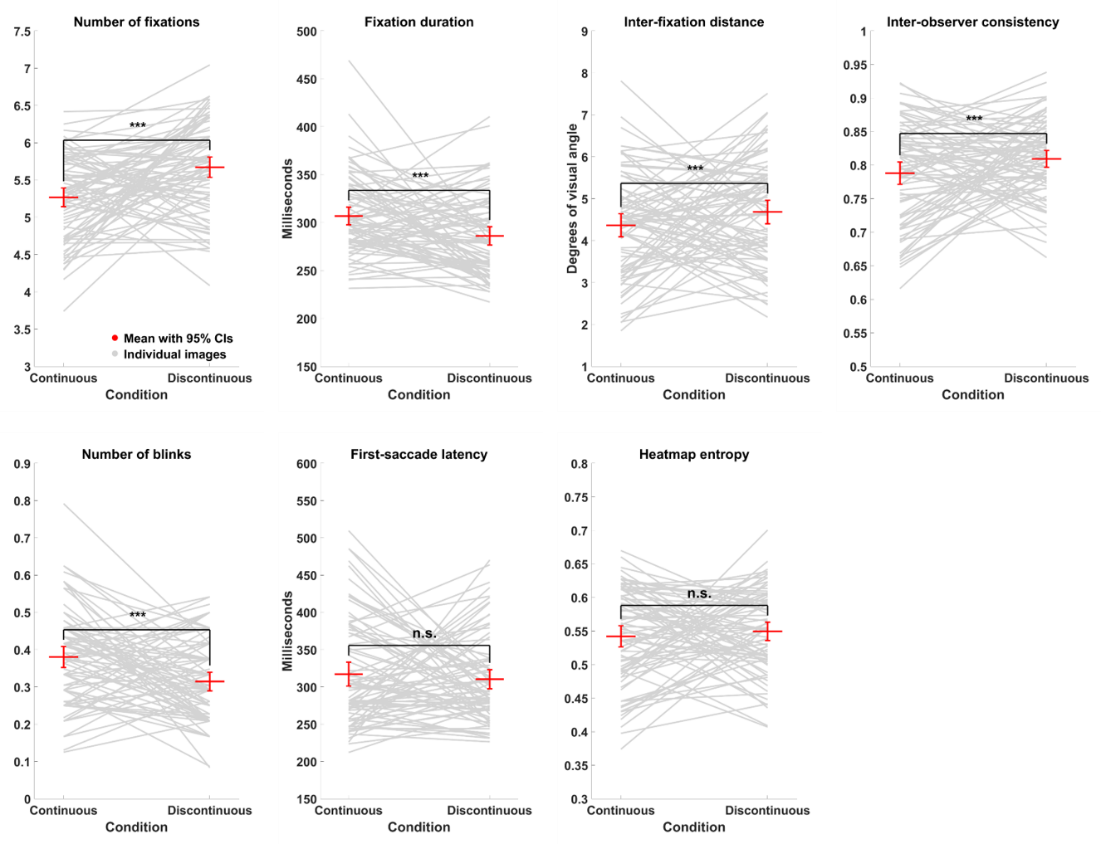


**Figure S2.**

*Results of Experiment 2*


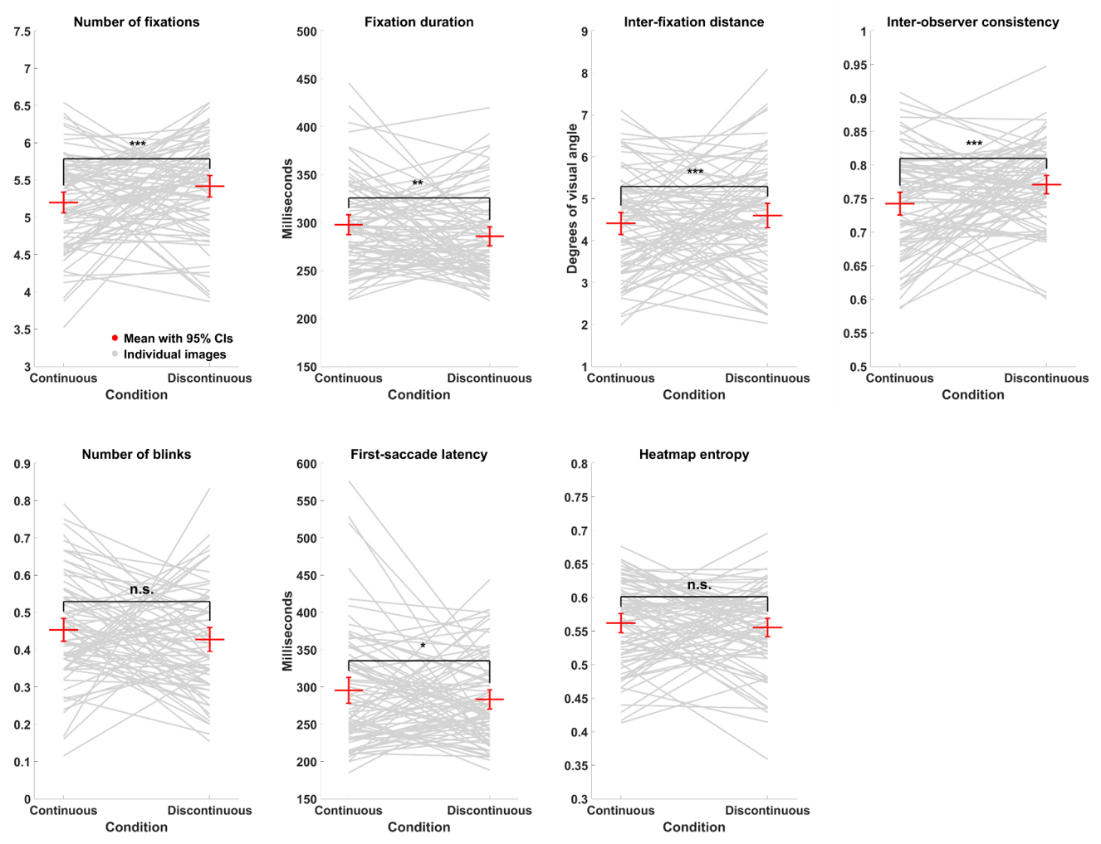


**Attention-check questions used in Experiment 1**

Listed below are ten sets of responses to the attention-check questions used in Experiment 1 and the links to the corresponding critical frames with which these responses were paired. The content of the questions was always the same (direct quote): ‘*Which description best matches the last image?*’. Response a) is always correct (but the responses were presented to participants in a randomized order). The full list of questions can be found in the preregistration, in the table allowing for downloading our stimuli.

https://thejar.hitchcock.zone/1000/Family%20Plot%20(1976)/0160.jpg

- 1. men in a room with a woman holding a gun
  2. a group of people in an elevator
  3. people walking down a hallway
  4. a group of men looking through a stack of documents

https://thejar.hitchcock.zone/1000/Topaz%20(1969)/0027.jpg

- 1. people walking down a street
  2. a man jogging in a park
  3. somebody walking a dog
  4. a boy flying a kite in a park

https://thejar.hitchcock.zone/1000/Marnie%20(1964)/0066.jpg

- 1. somebody wearing heels standing by a drain
  2. a child splashing in a puddle
  3. a snow-covered path
  4. a cat sleeping in a sunny room

https://thejar.hitchcock.zone/1000/North%20by%20Northwest%20(1959)/0039.jpg

- 1. a man checking his watch on a busy street
  2. people waiting at a crosswalk
  3. people standing in line to check in for a flight
  4. a man walking through a park

https://thejar.hitchcock.zone/1000/The%20Man%20Who%20Knew%20Too%20Much%20(1956)/0055.jpg

- 1. a group of people sitting on a bus talking
  2. a family having dinner together
  3. parents consoling their crying child
  4. people boarding an airplane

https://thejar.hitchcock.zone/1000/To%20Catch%20a%20Thief%20(1955)/0349.jpg

- 1. people playing a game of roulette
  2. a synchronized swimming competition
  3. people watching a fireworks display
  4. people watching the sun set

https://thejar.hitchcock.zone/1000/North%20by%20Northwest%20(1959)/0940.jpg

- 1. people talking in a house
  2. a family having dinner together
  3. friends exchanging Christmas presents
  4. people packing up their belongings

https://thejar.hitchcock.zone/1000/The%20Man%20Who%20Knew%20Too%20Much%20(1956)/0806.jpg

- 1. a woman entering a concert hall being spoken to by an usher
  2. a group of women getting their hair done in a hair salon
  3. people dancing at a party
  4. a woman making a speech at a wedding

https://thejar.hitchcock.zone/1000/Family%20Plot%20(1976)/0626.jpg

- 1. cars travelling down a road
  2. a tractor going down a country road
  3. a police car speeding down a road
  4. a deer in the middle of the road

https://thejar.hitchcock.zone/1000/Topaz%20(1969)/0593.jpg

- 1. two vehicles travelling down a road
  2. an airplane taking off
  3. a farmer ploughing a field
  4. a fruit stand on the side of the road

**‘Generic’ attention-check questions used in Experiment 2**

1. How many human faces were there? / None / 1 / 2 to 3 / 4 or more
2. Was the scene indoors or outdoors? / Indoor / Outdoor
3. Is this scene likely to be in a city? / Yes / No
4. How many people were sitting down? / None / 1 / 2 to 3 / 4 or more
5. How many people were standing up? / None / 1 / 2 to 3 / 4 or more
6. How many men were in the image? / None / 1 / 2 to 3 / 4 or more
7. How many women were in the image? / None / 1 / 2 to 3 / 4 or more
8. How many trees were in the image? / None / 1 to 3 / 4 or more
9. Were there any windows in the image? / Yes / No
10. Were the facial expressions of characters mostly: / Happy / Serious / Neutral / There were no faces or facial expressions were not visible
11. Was the scene in a public place? / Yes / No
12. Were there any cars in the image? / Yes / No
13. Was it day or night? / Day / Night / Could not tell
14. Were any of the characters holding an object? / Yes / No / There were no characters or could not tell
15. Were the majority of characters in the scene dressed for cold weather or warm weather? / Cold / Warm / There were no characters or could not see their clothing
16. Were the characters in the scene mostly aged: / Under 18 years old / 18-40 / Over 40 years old / There were no characters

**References**

Gameiro, R. R., Kaspar, K., König, S. U., Nordholt, S., & König, P. (2017). Exploration and Exploitation in Natural Viewing Behavior. *Scientific Reports*, *7*(1). https://doi.org/10.1038/s41598-017-02526-1

Krassanakis, V., Filippakopoulou, V., & Nakos, B. (2014). EyeMMV toolbox: An eye movement post-analysis tool based on a two-step spatial dispersion threshold for fixation identification. *Journal of Eye Movement Research*, *7*(1), 1–10. https://doi.org/10.16910/jemr.7.1.1

Lakens, D. (2014). Performing high-powered studies efficiently with sequential analyses. *European Journal of Social Psychology*, *44*(7), 701–710. https://doi.org/10.1002/ejsp.2023

Lyu, M., Choe, K. W., Kardan, O., Kotabe, H. P., Henderson, J. M., & Berman, M. G. (2020). Overt attentional correlates of memorability of scene images and their relationships to scene semantics. *Journal of Vision*, *20*(9), 2. https://doi.org/10.1167/jov.20.9.2

Nuthmann, A., & Einhäuser, W. (2015). A new approach to modeling the influence of image features on fixation selection in scenes. *Annals of the New York Academy of Sciences*, *1339*(1), 82–96. https://doi.org/10.1111/nyas.12705

Pahl, R. (2018). *GroupSeq: A GUI-based program to compute probabilities regarding group sequential designs* (1.3.5). http://cran.r-project.org/package=GroupSeq

Pocock, S. J. (1977). Group sequential methods in the design and analysis of clinical trials. *Biometrika*, *64*(2), 191–199. https://doi.org/10.1093/biomet/64.2.191
